# Physical Activity, Cardio-Respiratory Fitness, and Metabolic Traits in Rural Mexican Tarahumara

**DOI:** 10.1002/ajhb.22239

**Published:** 2012-02-05

**Authors:** Dirk Lund Christensen, Imelda Alcalá-Sánchez, Irene Leal-Berumen, Miguel Conchas-Ramirez, Soren Brage

**Affiliations:** 1 Department of International Health, University of CopenhagenCopenhagen, Denmark; 2 Steno Diabetes CenterGentofte, Denmark; 3 Facultad de Derecho, Universidad Autónoma de ChihuahuaChihuahua, México; 4 Facultad de Medicina, Universidad Autónoma de ChihuahuaChihuahua, México; 5 Facultad de Educacion Física, Universidad Autónoma de ChihuahuaChihuahua, México; 6 MRC Epidemiology UnitCambridge, United Kingdom

## Abstract

**Objectives:**

To study the association between physical activity energy expenditure (PAEE) and cardio-respiratory fitness (CRF) with key metabolic traits and anthropometric measures in the Tarahumara of Mexico.

**Methods:**

A cross-sectional study was carried out in five rural communities in Chihuahua, México including 64 adult Tarahumara, mean (SD) age 40.7 (12.9) years. Using a combined accelerometer and heart rate sensor, PAEE was measured over three consecutive days and nights and a sub-maximal step test was carried out in order to (1) calibrate heart rate at the individual level and (2) to estimate CRF. Random blood glucose level and resting blood pressure (BP) were measured with standard anthropometrics.

**Results:**

Mean (SD) PAEE was 71.2 (30.3) kJ kg^−1^ day^−1^ and CRF was 36.6 (6.5) mlO_2_ min^−1^ kg^−1^. Mean (SD) glucose was 127.9 (32.4) mg/dl, with 3.3% having diabetes. Mean (SD) systolic and diastolic BP was 122 (20.8) and 82 (14.8) mm Hg, respectively, with 28.1% having hypertension. Mean body mass index was 27.5 (4.2) kg m^−2^, with 71.9% being overweight. Following adjustment for age and sex, weak inverse associations were observed between PAEE and systolic BP (β = −0.20, *P* = 0.27) and diastolic BP (β = −0.16, *P* = 0.23); and between CRF and systolic BP (β = −0.51, *P* = 0.14) and diastolic BP (β = −0.53, *P* = 0.06). The inverse associations with glucose were also weak and not statistically significant for neither PAEE (β = −0.01, *P* = 0.63) nor CRF (β = −0.05, *P* = 0.27).

**Conclusions:**

This study suggests high levels of overweight and hypertension in the Tarahumara, and points to fitness and physical activity as potential intervention targets although findings should be confirmed in larger samples. Am. J. Hum. Biol. 2012. © 2012 Wiley Periodicals, Inc.

Metabolic disease and especially Type 2 diabetes is endemic in indigenous populations of North America with the US Pima as the prime example (Schulz et al., 2006). To what extent this is driven by physical activity is yet to be firmly established. Physical activity has mainly been assessed in epidemiological studies among Native Americans using questionnaires (Kriska et al., 1993), but this method has limitations and needs to be interpreted with caution.

A sedentary lifestyle has been shown to be an independent risk factor for metabolic disease (Slentz et al., 2009) and high levels of physical activity and cardio-respiratory fitness (CRF) are associated with a more favorable metabolic risk profile and lower mortality (Pedersen and Saltin, 2006). Early reports suggest high levels of physical activity and fitness in the Tarahumara of Mexico (Balke and Snow, 1965) but no recent data exist which include objectively measured physical activity and CRF and metabolic disease markers in this population.

This pilot study examined the association between habitual physical activity energy expenditure (PAEE) and CRF with key metabolic traits and anthropometric measures in Mexican Tarahumara, an indigenous population with a strong running tradition (Rascón and Batista, 1994) but undergoing transition and growing obesity rates (Monarrez-Espino and Greiner, 2000).

## METHODS

### Participants

We recruited 64 adult (24 men, 40 women) Tarahumara volunteers in a cross-sectional study carried out in October 2010. The study sites were the town of Guachochi, and the villages of Agua Zarka, Cabórachi, Kírare, and Tónachi in the Sierra Madre Occidental, Chihuahua, Mexico. The study sites are situated ∼ 1.150 to 2.400 meters above sea level. Inclusion criteria were permanent residency in one of the aforementioned study sites and written consent to participate.

### Procedures

In a subsample (not differing significantly from the larger sample by age, sex, body mass index (BMI), glucose, and blood pressure (BP)), physical activity was measured over 3 days and nights with a combined accelerometer and heart rate sensor (Actiheart, CamNtech Ltd, UK). The monitor was applied to the chest on two ECG electrodes (Brainclinics, Nijmegen, The Netherlands). CRF was derived as a *V*O_2max_ estimate using a standardized step test and PAEE modeled as described elsewhere (Brage et al., 2005; Brage et al., 2007).

Random capillary blood glucose test was determined by the glucose dehydrogenase method (HemoCue B-Glucose 201+, Ängelholm, Sweden). BP was measured using a BP oscillometric monitor (Omron HEM-412C, Kyoto, Japan) on the right mid-upper arm. Hypertension was defined as systolic BP = 140 and/or diastolic BP = 90 mm Hg.

BMI = 25 kg m^−2^ was used to define overweight, and waist circumferences (WC) = 80 and = 94 cm for women and men, respectively, were used to define central obesity (WHO, 2000). Arm muscle area and arm fat area in cm^2^ were derived from measures of triceps skinfold thickness and mid-upper arm circumference using formulas according to Frisancho (1990). Body fat percentage was measured by bioimpedance (Tanita Pro Body Composition Analyzer TBF-310-GS, Tokyo, Japan).

### Analysis

Statistical analyses were carried out with Stata IC version 11.2. Mean differences were determined using a two-sample *t*-test. We used random capillary blood glucose and systolic and diastolic BP as dependent variables, and linear regression to test for associations with PAEE and CRF. *P*-values <0.05 were considered significant. The study was approved by the Ethical Committee of Science in Chihuahua, Mexico (no. 0003925).

## RESULTS

**Fig. 1:**
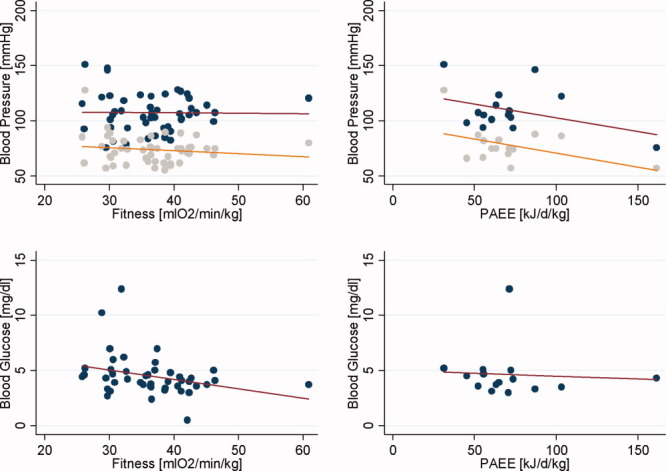
Associations between physical activity (*n* = 15), fitness (*n* = 48), and metabolic traits in adult Tarahumara. [Color figure can be viewed in the online issue, which is available at wileyonlinelibrary.com.]

For men and women combined, mean (SD) PAEE was 71.2 (30.3) kJ day^−1^ kg^−1^ and CRF was 36.6 (6.5) mlO_2_ min^−1^ kg^−1^. Mean (SD) glucose was 127.9 (32.4) mg dl^−1^, with 3.3% being above the threshold for diabetes. Mean (SD) systolic and diastolic BP was 122 (20.8) and 82 (14.8) mm Hg, respectively, with 28.1% having hypertension. Mean BMI was 27.5 (4.2) kg m^−2^, with 71.9% being overweight. Mean WC was 89.5 (11.2) cm and abdominal obesity was found in 68.8% of the participants. Mean (SD) body fat percentage was 30.7 (7.8) (Table 1). Following adjustment for age and sex, inverse associations between PAEE and systolic BP (β = −0.20, *P* = 0.27) and diastolic BP (β = −0.16, *P* = 0.23) as well as between CRF and systolic BP (β = −0.51, *P* = 0.14) and diastolic BP (β = −0.53, *P* = 0.06) were observed. Inverse associations with blood glucose for PAEE (β = −0.01, *P* = 0.63) and CRF (β = −0.05, *P* = 0.27) were found as well (Fig. 1).

**TABLE 1:** Characteristics by sex in adult Tarahumara (n = 64) in mean values (SD)

Measure	Men (*n* = 24)	Women (*n* = 40)	*P*-value
Age (years)	41.2 (11.6)	40.5 (13.8)	0.84
Weight (kg)	79.5 (10.9)	62.3 (11.1)	<0.001
Height (cm)	168.4 (6.8)	151.2 (6.1)	<0.001
Body mass index (kg m^−2^)	28.1 (3.8)	27.2 (4.4)	0.43
Waist circumference (cm)	97.0 (10.6)	84.9 (9.0)	<0.001
Arm fat area (cm^2^)	17.4 (6.6)	26.0 (10.1)	<0.001
Arm muscle area (cm^2^)	62.9 (11.1)	42.1 (8.1)	<0.001
Body fat (%)	25.5 (6.7)	33.6 (7.0)	<0.001
Blood pressure (mm Hg)			
Diastolic	85 (13)	80 (15)	0.15
Systolic	129 (13)	120 (23)	0.09
Blood glucose (mg dl^−1^)^a^	122.5 (34.2)	131.5 (30.6)	0.28
Physical activity (kJ day^−1^ kg^−1^)^b^	63.6 (23.7)	76.3 (34.3)	0.44
Cardiorespiratory fitness (mlO_2_ min^−1^ kg^−1^)^c^	39.6 (7.2)	34.1 (4.8)	0.002

a Random capillary sample.

b Sample size *n* = 15 (6 men, 9 women).

c Sample size *n* = 48 (22 men, 26 women).

## DISCUSSION

We observed a high percentage of participants with hypertension (28.1%), high PAEE (∼71 kJ kg^−1^ day^−1^), but moderately low CRF (∼37 mlO_2_ min^−1^ kg^−1^) in the Tarahumara, taking the mean age of this sample (∼41 years) into account. Further, we found weak inverse correlations between CRF and diastolic (borderline-significant) as well as systolic BP and similar but weaker correlations between PAEE and diastolic as well as systolic BP. This suggests that low CRF may be a risk factor for hypertension in the Tarahumara but the present study is underpowered to make any firm conclusions about the role of physical activity; this would require a larger sample, possibly combined with repeated periods of monitoring. The present study has, however, demonstrated that such measurements are feasible in this setting.

It is of note that the CRF range was wide (25.8–60.9 mlO_2_ min^−1^ kg^−1^). This indicates that in the rural Tarahumara the adverse consequences of lifestyle transition has made an impact on fitness, but also that high fitness can still be found; a 48-year-old man had the highest CRF in this sample.

In the Arizona Pima—closely related to the Mexican Pima from Sonora who are neighbors of the Tarahumara—prevalence of hypertension is low despite obesity and physical inactivity being endemic in this population. This has been attributed to a low sympathetic nervous system activity in combination with a reduced beta-adrenergic sensitivity (Tataranni et al., 1998). Given that geographical proximity rather than belonging to the same linguistic group correlates with genetic relationships (Rangel-Villalobos et al., 2000), i.e., the Tarahumara being genetically related to the Pima, the number of individuals with hypertension found in the present study was surprisingly high. However, the small number of Tarahumara participants (*n* = 64) could have introduced a sampling error.

In contrast to hypertension, we found few individuals with diabetes as well as non-significant inverse correlation between physical activity and fitness with blood glucose. When compared to the Mexican Pima, the low number of Tarahumara with diabetes was unexpected, as indigenous people of North America have been shown to be highly susceptible to diabetes (Yu and Zinman, 2007). However, very low prevalence of diabetes has been found as recently as the late 1990s among indigenous people in Mexico (Guerrero-Romero et al., 1997), which suggests that despite high genetic susceptibility the disease is driven by lifestyle changes (Schulz et al., 2006). Thus, the high PAEE found in the current study could very well be sufficient to protect the Tarahumara from diabetes. However, as with hypertension the small sample size in the present study is not sufficient to make any firm conclusions about diabetes prevalence in the Tarahumara population.

A large proportion of the study participants were overweight or obese when using standard WHO cut-offs. This supports an earlier study in the Tarahumara suggesting a growing obesity rate among adult women of reproductive age (Monarrez-Espino and Greiner, 2000). However, a recent study in Tarahumara children showed that obesity was uncommon indicating that unhealthy weight gain may be confined to adulthood in this population (Pena Reyes et al., 2009).

The current study is the first attempt to measure physical activity, CRF, and BP in Tarahumara men and women. Former investigations in the same population have mainly emphasized similar measurements in male runners due to their well-known athletic prowess (Groom, 1971). However, due to the small number of participants (*n* = 64) and even lower number who had physical activity (*n* = 15) and fitness (*n* = 48) measured, the results presented in this short report need to be interpreted with caution. A full-scale epidemiology study is warranted in order to obtain prevalence of diabetes and hypertension as well as the association between activity, fitness, and metabolic disease in the Mexican Tarahumara.
